# Simulating the spread of activation in neocortical circuits

**DOI:** 10.1186/1471-2202-12-S1-P209

**Published:** 2011-07-18

**Authors:** Samuel A Neymotin, Jason C Wester, Diego Contreras, William W Lytton

**Affiliations:** 1 Joint Biomedical Engineering Program SUNY Downstate/NYU-Poly, Brooklyn, NY, 11203, USA; 2 Dept. Neuroscience, University of Pennsylvania Medical School, Philadelphia, Pennsylvania, 19104, USA; 3 Dept. Physiology & Pharmacology, Biomedical Engineering, Neurology, SUNY Downstate, Brooklyn, NY 11203, USA; 4 Dept. Neurology, Kings County Hospital Center, Brooklyn, NY 11203, USA

## 

Dynamics in the brain are shaped by the connectivity of neocortical circuits and by incoming sensory information. The neocortical columnar architecture has been hypothesized to be a module for shaping dynamics to perform information processing. However, the dynamics and routing of information flow between elements of neocortical columns and amongst neocortical columns remains poorly understood.

Using NEURON[1] , we simulated a set of 11 horizontally connected layered neocortical columns which consisted of event-driven rule-based neurons wired according to known anatomical data and driven with random white-noise synaptic inputs [2]. We simulated afferent sensory inputs via the thalamus by strong stimulation of layer 4 pyramidal neuron AMPA/NMDA synapses in selected columns. To trace the role of different neocortical layers in information flow within and between columns, we simulated the focal application of the sodium channel blocker, tetrodotoxin (TTX), by inactivating selected layers of selected columns.

We tuned the network in order to achieve realistic cell firing rates and to avoid a high frequency of spontaneous population spikes. During baseline, a physiological frequency spectrum appeared as an emergent property, displaying dominant frequencies that were not present in either the inputs, or in the intrinsic or activated frequencies of any of the cell groups.

Thalamic stimuli evoked a brief population spike within layer 4. This population spike recruited recurrent activation within the column, evoking a set of 4-8 population spike discharges lasting from 500 - 1000 ms. The activity within the stimulated column spread in the classical neocortical column pathway: layer 4 -> layer 2/3 -> layer 5. After excitation reached layer 5, it spread to the neighboring columns by activating horizontal connections between layer 5 pyramidal cells. This activity then spread within the neighboring column from L5 to L2/3 pyramidal cells. After recurrent activation within L2/3, downward activation from L2/3 to L5 pyramidal cells initiated activity propagation from L5 to the next neighboring column. This activation sequence led to waves of population spikes gradually spreading outward from the initially stimulated column(s).

We found that selective blockage of layer 2/3 neurons via TTX simulation attenuated, but did not block the flow of activity between columns. Instead, activity was able to propagate from the stimulated L4 -> L5 pyramidal cells and then to neighboring columns via the L5 horizontal projections. When we blocked layer 5 neurons of stimulated columns, we found that activity was no longer able to flow from the stimulated to neighboring columns.

Our simulations demonstrate physiologically realistic spread of activation both within and between columns. Verifying the role of different layers in intra- and inter-columnar activation spread may be clinically relevant for containing seizures, where runaway excitation may be prevented by selective micro-ablation.
